# Enhancing pedicle screw placement training with a visual tracking guide template in augmented reality navigation

**DOI:** 10.3389/fmed.2026.1802255

**Published:** 2026-06-03

**Authors:** Peihai Zhang, Kai Zhang, Huiting Liu, Xuejun Yang

**Affiliations:** 1 Beijing Tsinghua Changgung Hospital, School of Clinical Medicine, Tsinghua Medicine, Tsinghua University, Beijing, China; 2 Peking Union Medical College Hospital, Beijing, China

**Keywords:** augmented reality, navigation, pedicle screw placement, training, visual tracking guide template

## Abstract

**Objective:**

This study presents a prototype visual tracking guide template (VTGT) for augmented reality (AR) navigation and assesses its initial feasibility for surgical training through a simulated pedicle screw placement exercise.

**Methods:**

The VTGT is used to complete the registration and real-time tracking of AR images. After wearing AR devices, junior residents simulate pedicle screw placement on spine models with K-wires. The navigation error is measured by assessing the deviation value of entry point (DVEP) and deviation value of trajectory angle (DVTA). The Clinical feasibility and safety were evaluated based on operation time, user experience, and the Gertzbein-Robbins scale.

**Results:**

Five junior residents participated in the experiment, and 100 K-wires were placed. The average time for each K-wire was 36.4 s, with an average user experience score of 87.8 points. The DVEP was 2.20 mm ± 0.55 mm, and the DVTA was 2.30° ± 0.78°. According to the Gertzbein-Robbins scale, all screws met the clinical safety requirements.

**Conclusion:**

This study demonstrates that the VTGT-based AR navigation technology is both feasible and useful for training junior residents in pedicle screw placement.

## Introduction

In recent years, spinal disorders, including disc herniation, spinal stenosis, scoliosis, and fractures, have grown increasingly prevalent, leading to a rising number of surgical interventions (1–4). Among these, pedicle screw placement is a fundamental yet technically demanding procedure in spinal surgery.

However, reliance on experience alone has been associated with a notably high screw misplacement rate, reported between 10 and 40% (5–11). Such inaccuracies carry significant risks, including serious iatrogenic injury and potential medical disputes (12–17). Therefore, ensuring the safety and accuracy of this procedure remains a critical challenge, especially for junior residents who are in the early stages of their surgical training.

Augmented reality (AR) has emerged as a promising navigational aid, capable of overlaying virtual guidance onto the real surgical field. Several studies have explored its application in pedicle screw placement (18–23). In our previous work, we introduced a visual tracking guide template (VTGT) to assist AR navigation during this procedure (24). The system utilized visible markers on the VTGT, which were tracked by the built-in camera of AR devices to achieve real-time spatial registration between virtual images and anatomical structures.

To explore its potential for surgical education, particularly in improving the anatomical understanding and hands-on skill acquisition of junior residents, we have now developed an upgraded VTGT. This iteration incorporates infrared reflective markers and an ergonomically optimized design, effectively addressing previous limitations such as interference from surgical lighting and physical obstruction of the operative field (19–21, 25). This technical innovation not only improves tracking reliability but also creates a simulated training environment that is less obstructed.

To evaluate the educational value of this enhanced AR navigation system, we conducted a structured training session in which trainees performed pedicle screw placement on simulated spine models using K-wires under its guidance.

## Method

Lumbar models were embedded in silicone gel based on the surgical scenario, and ten metallic spheres were fixed to each model for image registration. For the purpose of this training feasibility study, the setup was considered to provide a stable surgical field. CT scans were acquired and imported into a computer workstation to design a 3D path for the placement of pedicle screws. Ethical approval for this study was obtained from the Ethical Review Committee of Beijing Tsinghua Changgung Hospital, with approval number (24209-4-02). All participating residents gave written informed consent.

**Figure 1:**
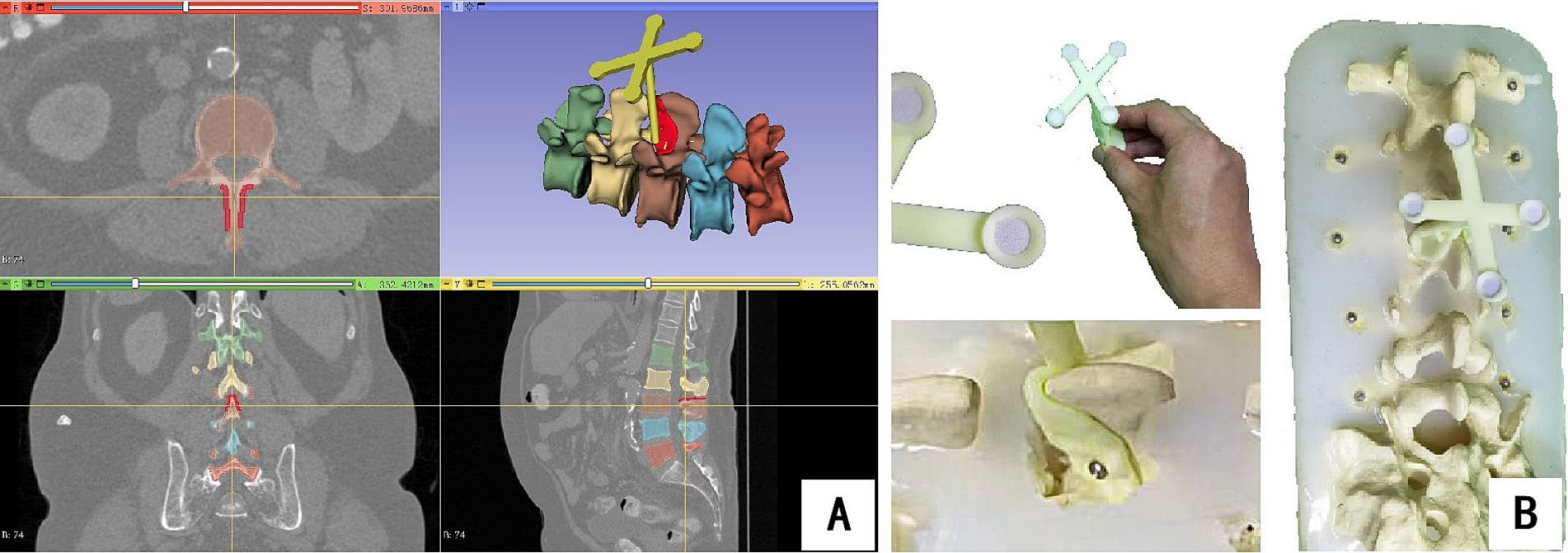
Design and installation of VTGT: **(A)** shows the design of VTGT, and **(B)** shows the appearance and installation.

The VTGT includes a base that was physically chimeric with the spinous process and a fixator that could be mounted with infrared reflective markers. The VTGT was designed to be vertebra-specific, and rigidly fixed to the L3 vertebra. The rigid connection between the VTGT and the spinous processes can promptly correct the errors caused by the movement of the model itself. Due to the unique structure of VTGT, the coordinate relation between the AR device and the model can be established. VTGT is produced by 3D printing, and the installation with the model is shown in Figure 1.

**Figure 2:**
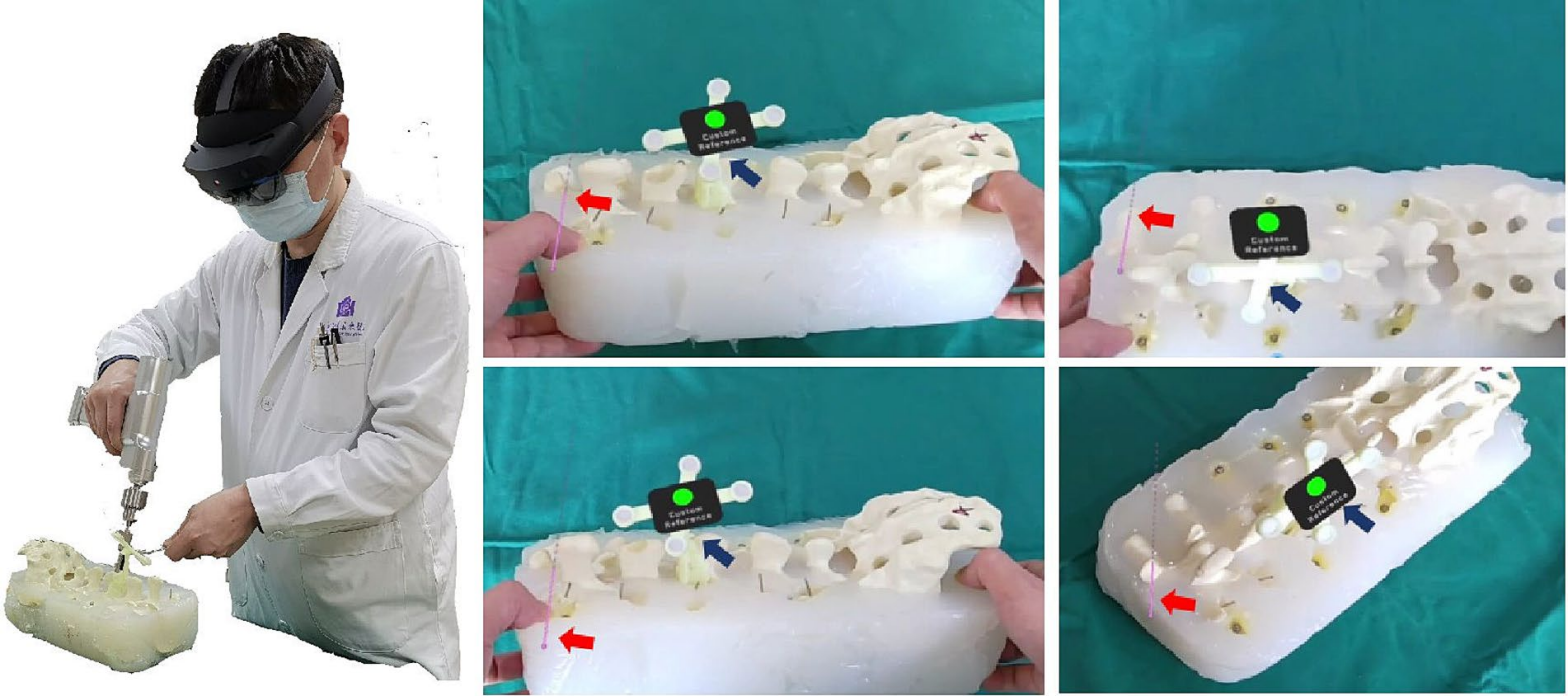
Pedicle screw placement with AR navigation: blue arrows show the VTGT, and red arrows show the virtual 3D path.

AR devices used in this study are the HoloLens2 (Microsoft, United States). K-wires were used for pedicle screw placement training. The entry points and trajectories required for the placement of the pedicle screws are integrated into virtual 3D paths that can be superimposed on the surgical site. Pedicle screw placement was performed on each model from L1 to L5 and a total of 100 K-wires were inserted. Five junior residents participated in the experiment, and two models were performed for each operator, as shown in Figure 2.

**Figure 3:**
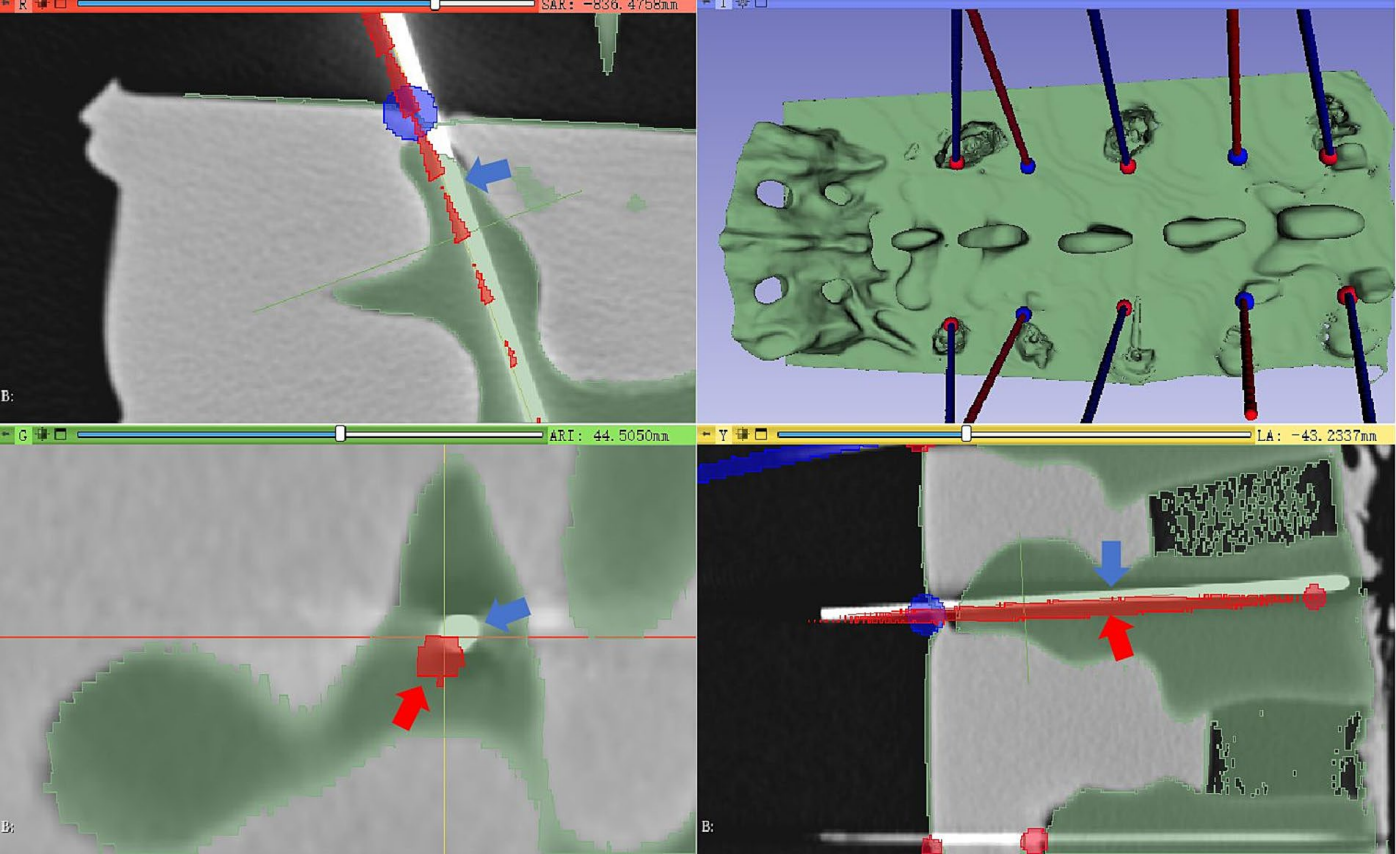
Registration of CT images: the blue arrow indicates the K-wire and the red arrow indicates the planned path.

After the operation, a CT scan was acquired. The image registration was completed using a preoperative CT scan, as shown in Figure 3. The distance between the actual and planned entry point was calculated, which was the DVEP. The angle between the long axis of the planned path and the long axis of the K-wire was calculated, which is the DVTA.

K-wire was expanded into a cylinder (6.0 mm*45 mm) to simulate a screw, and the distance between it and the outer wall of pedicle was calculated. Referring to the Gertzbein-Robbins scale (26), two senior doctors evaluated the clinical safety. If the pedicle was not broken or broken less than 2 mm, it was considered as a safe pedicle screw placement (grade A + B); otherwise, it was considered as a failure of pedicle screw placement.

In addition, the study conducted a questionnaire of time consumption and user experience. Based on acceptability and recognition, user experience evaluation was conducted, with a total of 100 points. The acceptability assessment encompasses factors such as the comfort of wearing the AR device, the potential for the AR image to create directional confusion, and the likelihood of users recommending it to their peers. The recognition assessment, on the other hand, focuses on whether AR navigation can expedite the surgical process and enhance surgical safety.

The statistical analysis of DVEP and DVTA were made, mean data were expressed as mean±standard deviation (Mean±SD). All tests were two-sided, *p* < 0.05 was considered as a significant difference. SPSS 26.0 was used for statistical analysis.

## Results

All five junior residents (R1-R5) successfully completed the pedicle screw placement training using AR navigation and 100 K-wires were inserted from L1 to L5. The mean DVEP for all models is 2.20 ± 0.55 mm, and 2.01 ± 0.60 mm for the resident 1 (R1), 2.32 ± 0.57 mm for the resident 2 (R2), as shown in Table 1.

**Table 1:** The DVEP of AR navigation.

Group	DVEP (mm)	Minimum (mm)	Maximum (mm)	Median (mm)	Numbers	*p*
R1	2.01 ± 0.60	0.95	3.15	2.15	20	>0.05
R2	2.32 ± 0.57	1.11	3.11	2.34	20	
R3	2.21 ± 0.53	1.41	3.45	2.11	20	
R4	2.11 ± 0.43	1.25	3.09	2.11	20	
R5	2.25 ± 0.55	1.57	3.33	2.44	20	
Total	2.20 ± 0.55	0.95	3.45	2.12	100	

The DVEP varied from 0.95 mm to 3.45 mm among residents, and there was no significant difference according to one-way ANOVA. The results above show that with the guidance of AR navigation, all residents can achieve good average positioning accuracy (less than 3.00 mm).

The DVTA varied from 0.60° to 5.59°, with an average of 2.30 ± 0.78°. The R1 had the largest mean DVTA (2.44 ± 1.00°), and the R3 had the smallest mean DVTA (2.16 ± 0.77°). There was no significant among different residents, as shown in Table 2.

**Table 2:** The DVTA of AR navigation.

Group	DVTA (°)	Minimum (°)	Maximum (°)	Median (°)	Numbers	*p*
R1	2.44 ± 1.00	1.14	5.59	2.18	20	>0.05
R2	2.19 ± 0.68	0.92	3.35	2.13	20	
R3	2.16 ± 0.77	0.58	3.69	2.13	20	
R4	2.34 ± 0.59	1.06	3.66	2.33	20	
R5	2.35 ± 0.75	0.72	3.67	2.32	20	
Total	2.30 ± 0.78	0.60	5.59	2.21	100	

The above results suggest that all residents can achieve good average DVTA (less than 3.00°), indicating that the directivity and stability of AR navigation system are reliable.

The operation time ranges from 695 s to 788 s, and all residents can complete one K-wire placement in less than 40 s on average. The user experience score ranged from 85 to 91 points, with an average score of 87.8 points, indicating that residents were satisfied with the system.

According to the Gertzbein-Robbins scale, all 100 screws were safe, 95 grade A screws were located completely inside pedicles, and five grade B screws broke through pedicles less than 2 mm, as shown in Table 3.

**Table 3:** Clinical safety assessment for pedicle screw placement.

Category		R1	R2	R3	R4	R5	Total
Number of screws		20	20	20	20	20	100
Grade A		19	20	20	18	18	95
Grade B	Above	0	0	0	0	0	0
	Inside	0	0	0	1	1	2
	Below	1	0	0	0	1	2
	Outside	0	0	0	1	0	1
Grade A screws ratio		19/20	20/20	20/20	18/20	18/20	95/100

## Discussion

The integration of AR into surgical training represents a significant advance over conventional learning methods. Unlike traditional approaches that rely on static images or video demonstrations, which often fail to convey spatial relationships and procedural nuance, AR creates an interactive, immersive learning environment. By wearing AR devices, trainees can view high-fidelity virtual guidance, such as screw trajectories and anatomical structures, seamlessly overlaid onto the real-world surgical field. This integrated visual feedback allows learners to maintain focus on the operative site without diverting attention to external screens, thereby overcoming a key limitation of earlier navigation systems (12–14).

Central to the effectiveness of any AR navigation system is the accuracy and reliability of image registration and tracking. While methods that reduce manual registration may save time, they often compromise precision, especially when anatomical shifts occur during surgery (27–29). In contrast, the use of firmly affixed artificial markers has been shown to provide the stability required for clinical applications (21, 23, 30–32). In this study, the visual tracking guide template (VTGT) was designed to streamline this process: installation was completed within minutes, without the need for intraoperative CT or complex image processing. The system projects a clear 3D hologram of the pedicle anatomy and planned screw path, offering trainees real-time, spatially accurate guidance on entry point and trajectory, which significantly reduces operational variability and error.

More importantly, this AR-based simulation platform represents a step toward replicating real surgical scenarios in a controlled setting. Trainees can practice pedicle screw placement repeatedly in a controlled yet realistic setting, accelerating skill acquisition and building procedural confidence. The educational value is further supported by the performance metrics observed in this study: the DVEP of 2.20 ± 0.55 mm and the DVTA of 2.30° ± 0.78°, indicating that the system achieves accuracy comparable to that of advanced intraoperative navigation technologies.

Beyond technical performance, this model offers profound practical advantages for surgical education. Unlike resource-intensive cadaveric training or passive video learning, the AR-VTGT platform is reusable, scalable, and cost-effective over time. It actively engages trainees through hands-on, interactive navigation, fostering deeper anatomical understanding and technical proficiency than traditional, less immersive teaching tools. In this study, trainees reported that the system was intuitive to use and expressed confidence in its potential to shorten the learning curve and improve operative efficiency.

However, trainees used K-wires, which lack the threaded profile and mechanical interaction of actual pedicle screws. Consequently, this training model did not capture critical surgical skills such as the tactile feedback of breaching the cortex, the need for sustained axial force to advance the screw, or the control of torque to prevent stripping or misalignment. While the use of K-wires allowed for an isolated evaluation of the AR system’s visual guidance accuracy, the transferability of these skills to real screw insertion remains unproven. Future iterations of this training platform should incorporate actual screw inserters and pedicle models with realistic bone density to provide a more comprehensive haptic and proprioceptive learning experience.

An expanding body of literature supports the integration of AR into medical training, highlighting its effectiveness in anatomy education and procedural simulation (33, 34). The positive user feedback and robust accuracy data from this study reinforce the viability of VTGT-based AR navigation as a valuable educational tool. By providing a safe, repeatable, and realistic training environment, this approach not only enhances surgical skill development for junior residents but also holds promise for standardizing training protocols and ultimately improving patient outcomes.

Several limitations of this study should be acknowledged regarding the fidelity of the experimental setup. First, while the VTGT provided vertebra-specific registration, the lumbar model embedded in silicone did not fully replicate the physiological intervertebral motion present in live patients. Although the semi-rigid silicone minimized gross movements, any undetected micro-motion could theoretically affect registration accuracy in a clinical setting. Therefore, the navigation accuracy reported here (DVEP 2.20 mm) represents the system’s performance under ideal, quasi-static conditions. Future studies utilizing dynamic phantoms or human cadavers are necessary to evaluate the system’s robustness against intervertebral movements.

## Conclusion

Our results confirm the feasibility and accuracy of the VTGT-based AR navigation in simulated surgical scenarios, highlighting its potential as an early-stage training adjunct. Further studies in dynamic models are required to establish its effectiveness in improving operative competence. Although the study involved a limited number of participants and did not fully evaluate cost, hardware, and staffing implications, it serves as a promising proof of concept for further development in structured surgical education.

## Data Availability

The raw data supporting the conclusions of this article will be made available by the authors, without undue reservation.
